# Effects of the stress hormone norepinephrine on the probiotic properties of *Levilactobacillus*: antibacterial colonization, anti-inflammation, and antioxidation

**DOI:** 10.3389/fmicb.2025.1526362

**Published:** 2025-02-10

**Authors:** Lingdi Niu, Mingchun Gao, Yifan Li, Chaonan Wang, Chuankun Zhang, Haoyuan Duan, Hai Li, Fang Wang, Junwei Ge

**Affiliations:** ^1^Heilongjiang Provincial Key Laboratory of Zoonosis, College of Veterinary Medicine, Northeast Agricultural University, Harbin, China; ^2^State Key Laboratory for Animal Disease Control and Prevention, Harbin Veterinary Research Institute, Chinese Academy of Agricultural Sciences, Harbin, China

**Keywords:** *Levilactobacillus*, catecholamine hormone, antimicrobial activity, probiotic effects, microbial endocrinology

## Abstract

Probiotics as antibiotic alternatives are unstable for use under stress in clinical applications. To explore the influence of catecholamine hormones on probiotic bacterial inhibition and antimicrobial activity, we tested the effects of norepinephrine (NE) on *Levilactobacillus in vitro* and in a mouse model. The *in vitro* results showed that in the presence of NE, 80% of *Levilactobacillus* strains showed increased growth rate and more than 80% of the strains indicated lower antimicrobial activity at 22 h. Furthermore, in the mouse model, NE weakens the protective effect of *L. brevis* 23,017 on *Escherichia coli* infection, which is shown by the decreased ability of antibacterial colonization, antioxidation, and anti-inflammation, and downregulating the expression of antioxidant genes and intestinal mucosal barrier-related genes. At the same time, the addition of NE modulates the bacterial microbiota richness and diversity in the intestine, disrupting the balance of intestinal probiotics. These findings provide evidence that NE reduces the probiotic ability of *Levilactobacillus* and illustrates the plasticity of the probiotics in response to the intestinal microenvironment under stress.

## 1 Introduction

In 1992, Lyte and Ernst introduced the concept of “microbial endocrinology” to assess the effect of bacterial growth under stress hormones, thus showing the two-way interaction between microorganisms and humans’ neuroendocrine factors (Lyte and Ernst, 1992). In past research, stress could increase neuroendocrine hormones, particularly glucocorticoids and catecholamines (Felten and Olschowka, 1987; Lymperopoulos et al., 2008; Webster Marketon and Glaser, 2008). Catecholamine hormones, including epinephrine, norepinephrine (NE), and dopamine, can affect the growth of many pathogenic bacteria *in vitro* (Coulanges et al., 1997; Nakano et al., 2007; Sandrini et al., 2014; Gao et al., 2019). Some studies have also shown that the virulence and biofilm formation of bacteria, such as *Salmonella* (Hiller et al., 2019) and *Escherichia coli* (*E. coli*) (Vlisidou et al., 2004), can be regulated by catecholamines, and this affects the outcome of infection by these bacteria on numerous hosts. However, there are few reports on the effect of catecholamine hormones on probiotics. Combined with proteomic analysis, Scardaci et al. assessed that *in vitro* norepinephrine (NE) treatment of *Enterococcus faecalis* enhanced the abundance of proteins involved in adhesion and immune stimulation (Scardaci et al., 2021). Cambronel et al. (2020) proposed that in *E. faecalis*, catecholamine hormones can promote adherence to eukaryotic cells and biofilm formation, and structural modeling and molecular docking confirmed that *E. faecalis* contains adrenergic sensors that interact with epinephrine and NE. However, the aforementioned studies were only based on bioinformatics analysis and *in vitro* tests to investigate the molecular level or biofilm and adhesion of probiotics.

Some researchers have also conducted a few *in vivo* experiments. Dong et al. discovered that NE increased the ability of *Aeromonas hydrophila* to proliferate in the lungs of mice (Dong et al., 2016). Some studies have also shown that catecholamine hormones can increase the pathogenicity of *Salmonella* in mouse, chicken, and bovine models (Williams et al., 2006; McCuddin et al., 2008; Methner et al., 2008; Pullinger et al., 2010b; Pullinger et al., 2010a), *Yersinia ruckeri* toward rainbow trout (Torabi Delshad et al., 2019), *A. hydrophila* in crucian (Gao et al., 2019), *Vibrio harveyi* and *Vibrio campbellii* in juvenile shrimp (Pande et al., 2014; Yang et al., 2014), and *Pseudomonas aeruginosa* toward Galleria mellonella larvae (Cambronel et al., 2019). Nevertheless, these studies have only looked at pathogenic microorganisms, and no scholars have yet explored the effect of catecholamine hormones on probiotics *in vivo*. We hypothesize that this interaction of host signals with *Levilactobacillus* strains may affect their bacterial properties and influence their probiotic effects. A better understanding of microbial endocrinology in the field of probiotics will allow a more comprehensive interpretation of how stress hormones are involved in the colonization of the microbiota and the ecological balance of the gut or other organs, which may help in the development of new therapies with medical and economic benefits.

In our research, we tried to evaluate the effect of NE on the probiotic properties *Levilactobacillus* based on the body injury caused by *E. coli*, which could potentially provide ideas to improve the unstable application effects of *Levilactobacillus* as antibiotic alternatives. For this purpose, (i) we simulated the internal environment (serum-SAPI medium) *in vitro* to investigate the effects of NE on the growth rate, viable bacteria count, bacterial inhibition capacity, and acid production capacity of *Levilactobacillus* strains; (ii) we selected the most suitable probiotic *Levilactobacillus* strains for investigating the effect of NE on the probiotic properties of *Levilactobacillus* in a mouse model; (iii) we established a BALB/c mouse model with *E. coli* standard strain infection and *Levilactobacillus* treatment; and (iv) we observed the intestinal pathological sections, oxidative damage, expression of inflammatory factors, intestinal mucosal barrier-related factors, Nrf2 and TLR4 and their downstream genes, and microbial amplicon sequencing and biological information analysis. Our findings shed light on the involvement of stress-related hormones in probiotics’ positive effects on the organism.

## 2 Materials and methods

### 2.1 Bacteria and medium

The *E. coli* CVCC230 strain was purchased from the China Veterinary Drug Supervision Institute. Nalidixic acid was used to induce the CVCC230 strain to be resistant to 50 μg/mL nalidixic acid with the method in the reference (Daudelin et al., 2011). *Levilactobacillus brevis* L5, 23010, 23017, 27197, 27053, 21060, 27058 and 23027 were isolated by our laboratory (Cui et al., 2018; Shi et al., 2019). As in almost all previous studies, the serum-SAPI medium was used in this study, which simulates the internal environment of the host (Lyte and Ernst, 1992; Freestone et al., 1999; Bearson et al., 2008; Li et al., 2009; Inaba et al., 2016).

### 2.2 Growth of *Levilactobacillus* strains

The effect of NE (Sigma-Aldrich, USA) on the growth of *Levilactobacillus* strains was carried out according to the method of Inaba et al. (2016). The bacteria were cultivated in serum-SAPI medium in a ratio of 1:100 as the *Levilactobacillus* groups and those added to NE (100 μM) as the *Levilactobacillus* + NE groups. We selected 100 μM NE for *in vitro* testing based on the concentrations in reference (Cogan et al., 2007; Gao et al., 2019; Scardaci et al., 2021). The bacterial suspension was collected at time periods of 2 h, 4 h, 6 h, 8 h, 12 h, 24 h, 28 h, 32 h, and 48 h. At the same time, live bacterial counts were also recorded.

### 2.3 Antimicrobial activity of *Levilactobacillus* strains

The antimicrobial activity of NE on *Levilactobacillus* strains was evaluated using an agar-well diffusion test, as described in the reference (Shi et al., 2019). The *Levilactobacillus* culture solution and 100 μM NE were added to the serum-SAPI medium as the experimental group, and the *Levilactobacillus* culture solution alone was used as the control group. The antimicrobial activity was assessed against *E. coli* (CVCC230), *Staphylococcus aureus* (CVCC26003), and *Pseudomonas aeruginosa* (ATCC9027).

### 2.4 Co-culture of probiotic and pathogenic bacteria

The co-culture of the probiotic *L. brevis* 23,017 and pathogenic bacteria *E. coli* CVCC230 was carried out according to Yang et al. (2015), with slight modifications. They were suspended in sterile phosphate-buffered saline (PBS) before being co-inoculated in the serum-SAPI medium at a cell density of 1 × 10^6^ CFU/mL and incubated aerobically for 24 h at 37°C. The samples were then serially diluted and plated on eosin-methylene blue (EMB) agar containing 50 μg/mL nalidixic acid to enumerate the number of pathogen colonies. EMB medium is a selective medium for *E. coli*, which appears as purple-black colonies with a green metallic luster when *E. coli* cultured in EMB medium.

### 2.5 Acid production capacity of *Levilactobacillus* strains

The titratable acidity of the culture supernatant was determined by acid–base titration, following the methods described in reference (Navrátilova et al., 2022). The culture supernatant was collected at time periods of 2 h, 4 h, 6 h, 8 h, 12 h, 24 h, 28 h, 36 h, and 48 h. Hydrogen peroxide production was determined according to Eschenbach et al. (1989).

### 2.6 Animal experimental design

The Ethical Committee of the Institute approved all scientific experiments. All applicable international and national guidelines for the care and use of animals in experiments were followed. Approval (NEAUEC-20, 3 March 2020) was obtained from the Institutional Committee of Northeast Agricultural University for animal experiments. SPF BALB/c female mice (6–8 weeks of age) were purchased from the Laboratory Animal Centre, the Second Affiliated Hospital of Harbin Medical University (Harbin, China). The mice were maintained in a controlled environment and had free access to rodent food and tap water during a 12-h cycle of light and darkness.

In order to select the concentration and route of administration of NE, we first performed pre-experiments. The mice were divided randomly into five groups (*n* = 5/group), and they were vaccinated orally five different concentrations of NE, 16.9 mg/kg/day (100 μM), 8.45 mg/kg/day (50 μM), 4.23 mg/kg/day (25 μM), 1.69 mg/kg/day (12.5 μM), and 0 mg/kg/day (0 μM). The mice were kept under observation and the disease activity index (DAI) was recorded every 6 h and the NE administration route that causes the most significant clinical symptoms was identified. DAI is the sum of individual scores recorded for blood stool, stool consistency, and weight loss (Shinde et al., 2019). According to the method in literature (Cooper et al., 1993), the body weight of mice was recorded twice a day at the same time, and the average value was taken. In brief, scores were determined by reference.

**Figure 1:**
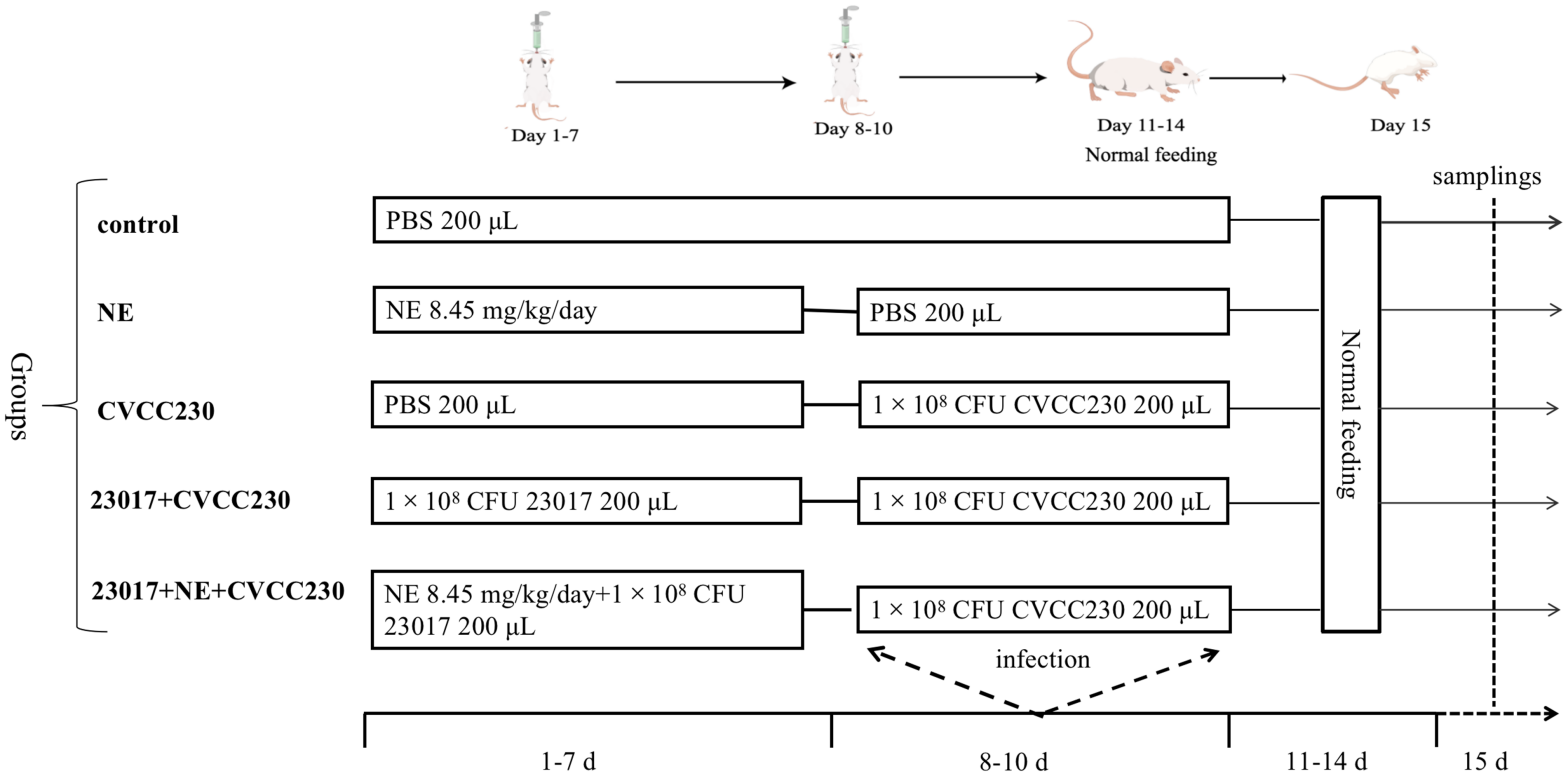
The infection model of the mice.

Based on the previous *in vitro* test data, we selected *L. brevis* 23,017 for animal testing. Before bacterial inoculation, the animals were given 0.25 mL of a 0.2 mol/L sodium bicarbonate solution. A total of 25 mice were randomly divided into 5 groups (5 mice in each) in a completely random design. Dietary treatments included the control group with oral PBS, the NE group with oral NE, the CVCC230 group with oral *E. coli* CVCC230, the 23,017 + CVCC230 group with oral *L. brevis* 23,017 and *E. coli* CVCC230, and the 23,017 + NE + CVCC230 group with oral *L. brevis* 23,017 cultured in the medium containing NE and *E. coli* CVCC230. The specific mouse infection model is shown in Figure 1. After 15 days of treatment, the mice in each group were sacrificed and used for the subsequent analysis. The blood was collected from the eyeballs, and the serum was separated and stored in a − 70°C freezer for further analysis. The duodenum tissues were immediately collected for experiments and fixed in 10% formalin for histological evaluation. The small intestine was collected for biological information analysis.

### 2.7 Bacterial enumeration

The bacterial enumeration of the duodenum was determined by the modified method of Nour et al. (2021). The entire duodenum was sampled for postmortem clinical assessment of parameters. The EMB containing 50 μg/mL nalidixic acid was used for counting agar.

### 2.8 Histopathology

According to the previous pathological tissue staining protocol (Trajković et al., 2007), the tissues were sectioned and images were obtained at 100× and 400× magnifications, respectively.

### 2.9 Antioxidant capacity

The antioxidant capacities of duodenum were determined using assay kits according to the manufacturer’s instructions (Nanjing Jiancheng Bioengineering Institute, Nanjing, China). The activities of superoxide dismutase (SOD), glutathione peroxidase (GSH-Px), and total antioxidant capacity (T-AOC) were expressed as U per mg protein. The activities of malondialdehyde (MDA) were expressed as nmol per mg protein.

### 2.10 Enzyme-linked immunosorbent assay (ELISA)

The levels of interleukin-6 (IL-6), interleukin-10 (IL-10), interleukin-1*β* (IL-1β), and myeloperoxidase (MPO) were measured via an enzyme-linked immunosorbent assay (ELISA) kit according to the manufacturer’s instructions (Boster Biological Technology, China).

### 2.11 Real-time quantitative polymerase chain reaction (PCR)

Based on the method of Gao et al. (2019), the detection of intestinal mucosal barrier-related functional genes was implemented with a slight modification. The RNA was extracted from the duodenum tissue using an RNA extraction kit (TransGen Biotech, Beijing, China) and then reverse-transcribed into cDNA using ReverTra Ace® (TOYOBO, Shanghai, China) following the manufacturer’s protocol. The cDNA was used for quantification and expression of various genes, including inflammation-related genes, Nrf2, and their downstream genes and intestinal mucosal barrier-related genes. Furthermore, the real-time quantitative PCR reactions were carried out using the Applied Biosystems® 7,500 Real-Time PCR System (Analytik Jena AG, Germany) according to the manufacturer’s instructions. Actin β-action was taken as the reference gene. Primers for this study were synthesized with the company (Comate Bioscience Co., Ltd., Jilin, China). The primer sequence sets used are provided in Supplementary Table S1. The relative mRNA levels were quantified using the 2^−ΔΔCt^ method (Livak and Schmittgen, 2001).

### 2.12 Microbial diversity analysis

Fecal pellets and contents of small intestine were collected from mice in different groups, and the microbial amplicon sequencing was conducted by Genesky Biotechnologies Inc. (Shanghai, China). Alpha diversity was used to analyze the complexity of species diversity in each sample, which included Chao1, ACE, Shannon, and Simpson.

### 2.13 Statistical analysis

All the data are expressed as ±standard error of the mean. The SPSS 22.0 software (SPSS Inc., Chicago, IL, USA) was utilized for statistical analyses. The one-way ANOVA with the least significant difference (LSD) post-hoc test was used to identify the significant differences between groups. *p* < 0.05 was considered to be statistically significant.

## 3 Results

### 3.1 NE promotes the growth and viable bacteria of *Levilactobacillus* strains

**Figure 2:**
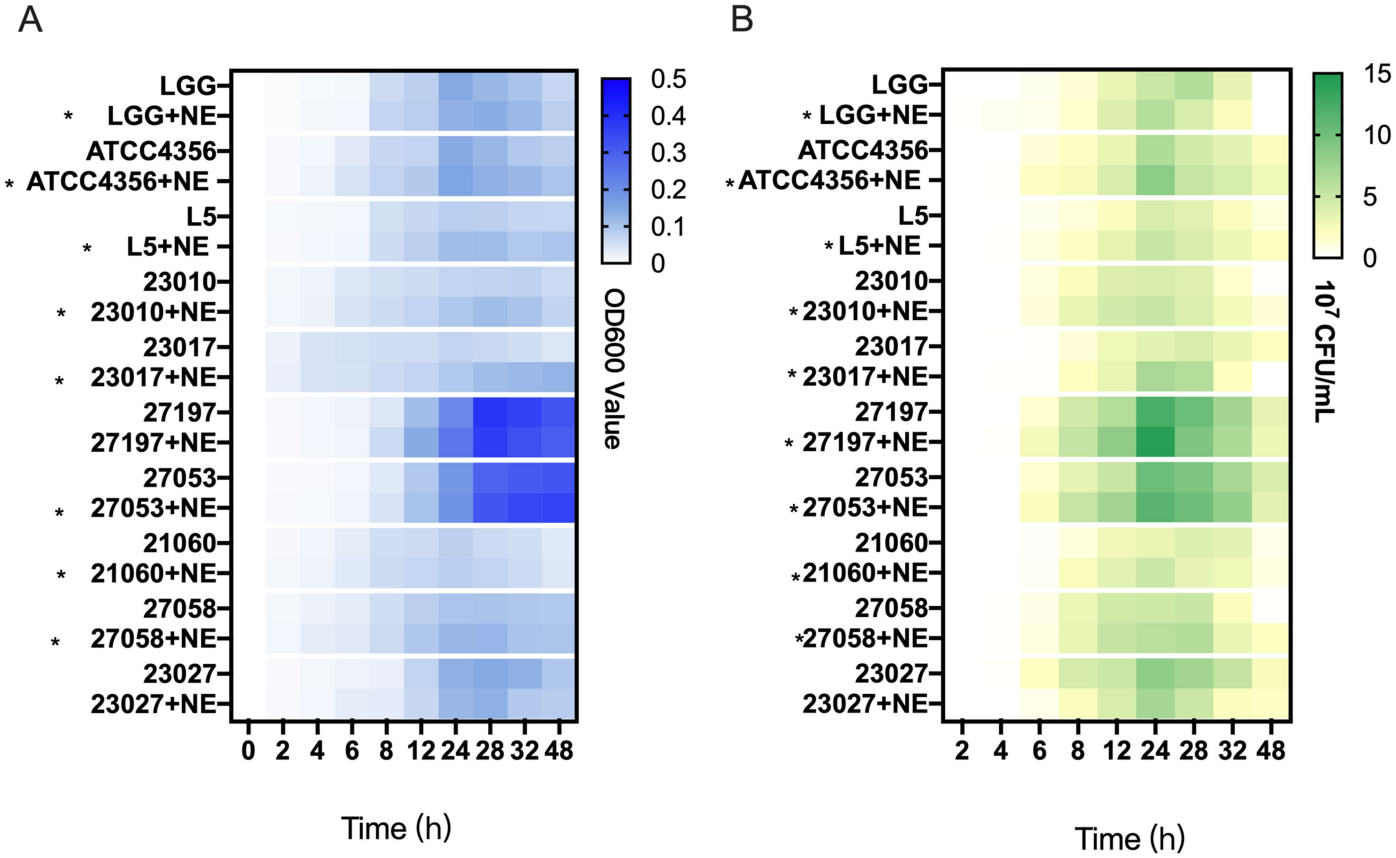
The effect of norepinephrine (NE) on the growth of *Levilactobacillus.* **(A)** Heatmap representing the OD600 value of *Levilactobacillus* growth within 0–48 h. Asterisks indicate strains with NE enhanced growth at 0–48 h. **(B)** Heatmap of the number of viable bacteria of *Levilactobacillus* growth within 0–48 h, the unit is colony-forming units per milliliter (10^7^ CFU/mL). Asterisks indicate strains with NE enhanced growth at 0–24 h.

To investigate whether NE has an effect on the growth of *Levilactobacillus* strains, we examined the growth rate and viable bacterial counts in ten strains of *Levilactobacillus in vitro*. In 0–48 h, the addition of NE resulted in an increase in the final culture density of 8 (80%) *Levilactobacillus* strains over control cultures (Figure 2A; Supplementary Figure S1) in addition to the 27,197 and 23,027 groups.

According to Figure 2B and Supplementary Figure S2, the growth trend of the average *Levilactobacillus* viable count is similar to the trend of the bacterial growth curve. In 0–24 h, the addition of NE resulted in an increase in the viable count of 9 (90%) *Levilactobacillus* strains over control cultures. In general, the results showed that NE has a promotive effect on the growth of most *Levilactobacillus* strains.

### 3.2 NE reduces the antimicrobial activity of *Levilactobacillus* strains

**Figure 3:**
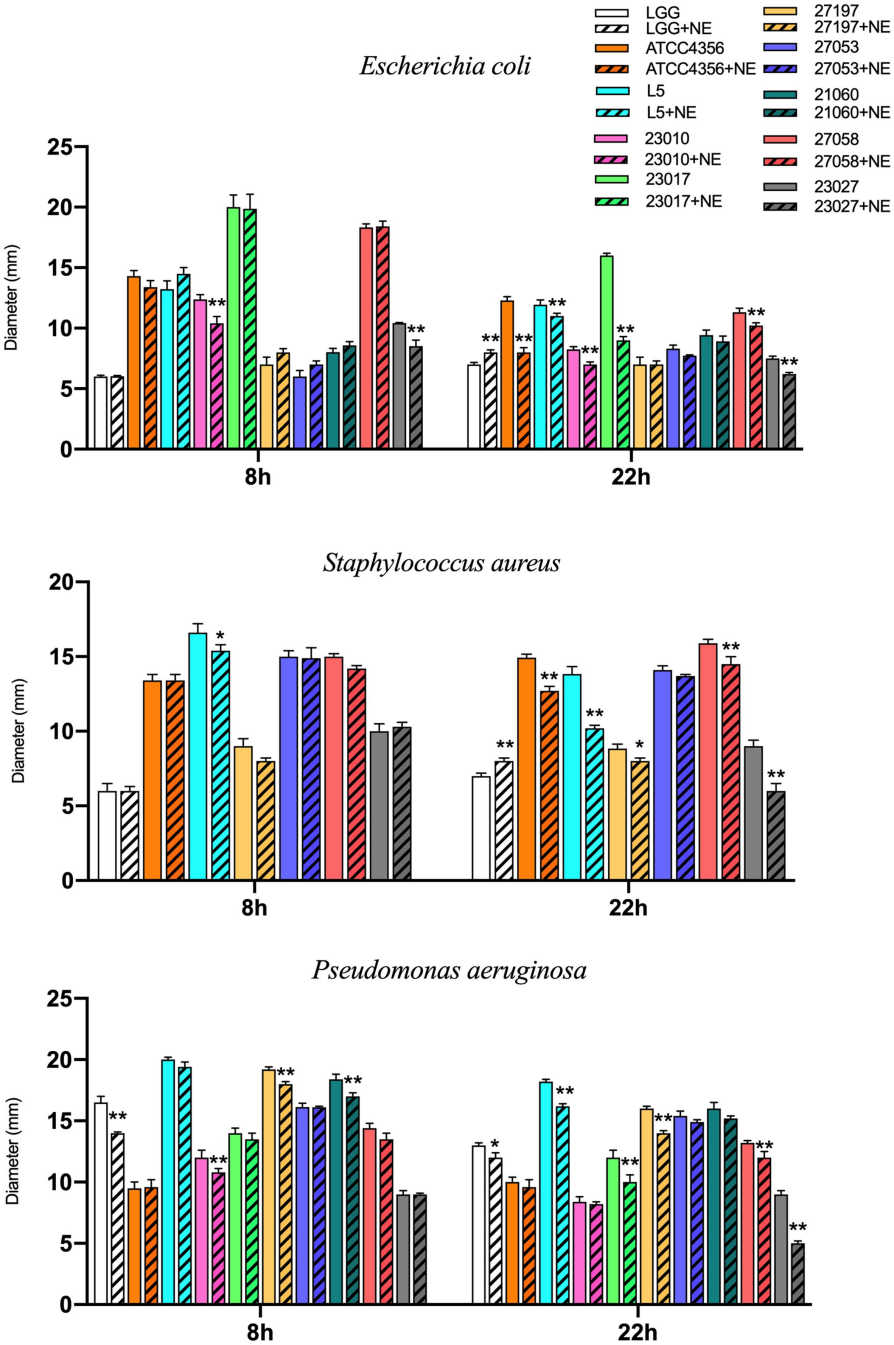
Effect of NE on antibacterial ability of *Levilactobacillus* to *E. coli, Staphylococcus aureus and Pseudomonas aeruginosa* in 8 h and 24 h. Different colors represent different strains, blank columns represent *Levilactobacillus* acted alone, and columns with slashes represent *Levilactobacillus* groups acting with NE. Experiments were repeated three times, *, **, respectively, represent significant differences (*p* < 0.05), significant differences (*p* < 0.01).

To explore the effect of NE on the inhibitory effect of probiotic *Levilactobacillus* strains on *E. coli*, *S. aureus*, and *P. aeruginosa in vitro*, we examined their antimicrobial activity at 8 h and 22 h. As shown in Figure 3, at 8 h for the *Levilactobacillus* strains tested, NE inhibited the antimicrobial activity of 3 (30%) strains against *E. coli*, with 2 of them being significantly inhibited; NE inhibited 3 (43%) strains against *S. aureus*, with 1 of them was significantly inhibited; and NE inhibited 7 (70%) strains against *P. aeruginosa*, with 4 of them being significantly inhibited. At 22 h, NE inhibited the antimicrobial activity of 8 (80%) *Levilactobacillus* strains against *E. coli*, with 6 of them being significantly inhibited; NE inhibited 6 (86%) *Levilactobacillus* strains against *S. aureus*, with 5 of them being significantly inhibited; and NE inhibited the 10 (100%) *Levilactobacillus* strains against *P. aeruginosa*, with 6 of them being significantly inhibited. In summary, the addition of NE reduced the antimicrobial activity of the majority of the *Levilactobacillus* strains.

### 3.3 NE increased the number of viable *Escherichia coli* in co-culture

**Figure 4:**
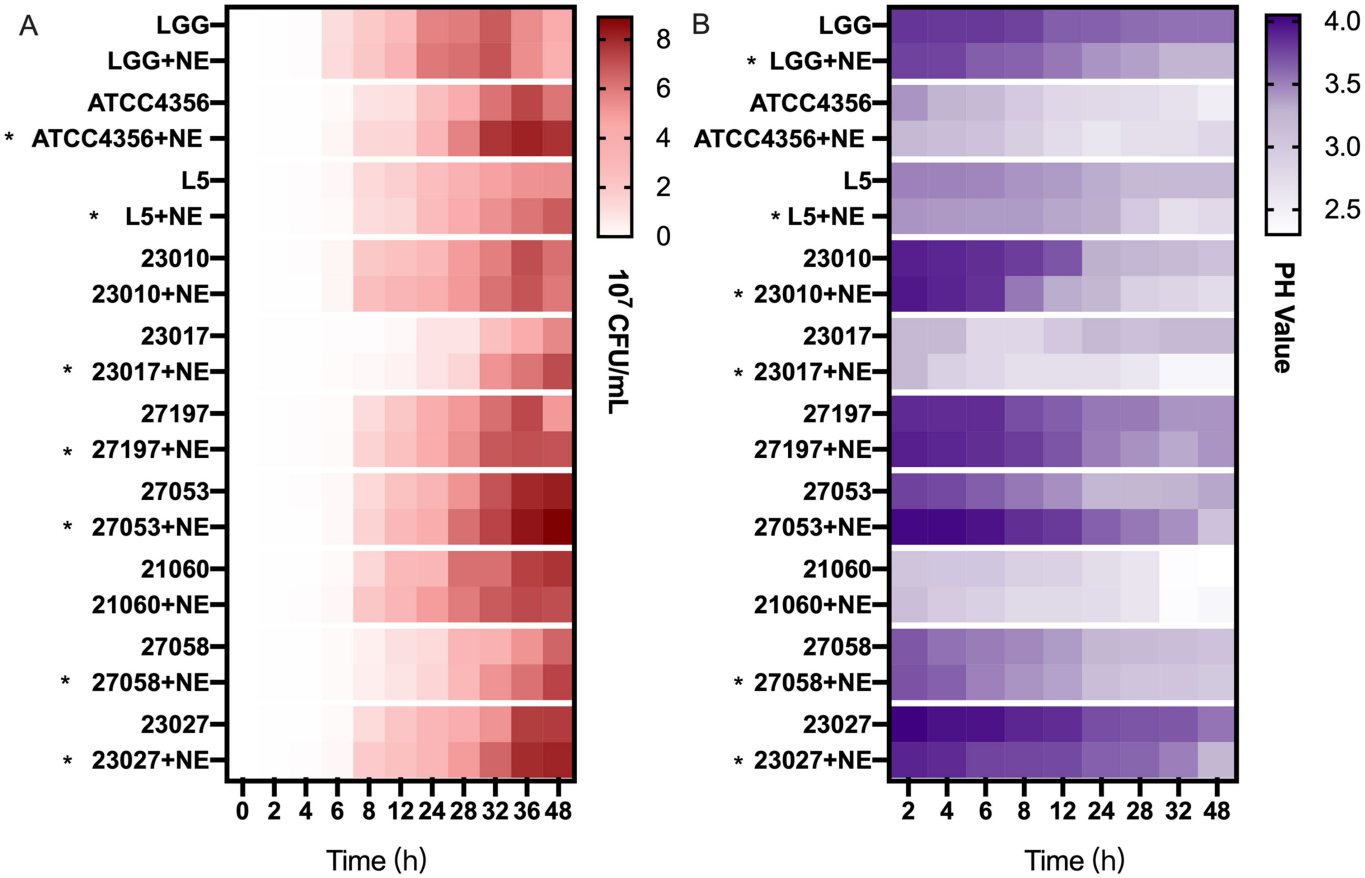
The effect of NE on the antimicrobial and acid production capacity of *Levilactobacillus*. **(A)** Effect of NE on the number of viable *Escherichia coli* (*E. coli*) bacteria under co-culture conditions, heatmap of the number of viable bacteria of *E. coli* (10^7^ CFU/mL). Asterisks indicate that co-culture with NE enhanced the number of viable bacteria of *E. coli*. **(B)** Effect of NE on acid production of *Levilactobacillus*, heatmap of the pH values of culture supernatant. Asterisks indicate that co-culture with NE enhanced the acid production capacity.

To simulate the intestinal environment in which the three factors NE, *L. brevis* 23,017, and *E. coli* CVCC230 coexist in the intestine and to investigate the effect of catecholamine on the mixed bacteria, we performed co-culture experiments. Figure 4A and Supplementary Figure S3 depict the viable counts of *E. coli*. In the early phase (0–24 h), in most groups (50%), mixed cultures were higher than controls, and this proportion increased to 70% after 24 h. Overall, in the co-culture system, the addition of NE increased the number of viable *E. coli*.

### 3.4 NE promotes the acid production capacity of the 10 *Levilactobacillus* strains

To investigate the reason why NE reduces the antimicrobial activity of *Levilactobacillus* strains, we examined their acid production capacity. As shown in Figure 4B and Supplementary Figure S4, for 60% of the tested strains, *Levilactobacillus* strains grown with NE generated higher concentrations of acid than those grown without it. However, for the ATCC4356, 27,197, 27,053, and 21,060 strains, the capacity of acid production was similar with or without NE. The addition of NE did not affect the ability of *Levilactobacillus* strains to produce hydrogen peroxide (data not shown).

### 3.5 NE reduces the ability of *Levilactobacillus brevis* 23,017 in inhibiting intestinal colonization by pathogenic bacteria

Mice orally administered with 8.45 mg/kg/day (50 μM) NE showed depression and loss of appetite within 48 h. Symptoms were relieved after 72 h, and normal diet was resumed. The mice in the high-concentration group of 16.9 mg/kg/day (100 μM) showed depression, trembling bodies, no eating or drinking, loss of appetite, weight loss, and even death within 72 h. Therefore, the 8.45 mg/kg/day (50 μM) concentration of NE was considered optimal.

To investigate whether NE regulates the competition between *Levilactobacillus* and pathogenic bacteria for colonization sites, we examined the viable *E. coli* count. The viable *E. coli* count was recorded as log10 CFU/g. *E. coli* CVCC230 was not detected in the livers of each group of mice. In duodenum tissues, the 23,017 + CVCC230 (6.55 ± 0.19) group and the 23,017 + NE + CVCC230 (7.15 ± 0.13) group significantly reduced the number of *E. coli* compared to the CVCC230 group (7.32 ± 0.12), while the number of *E. coli* increased in the 23,017 + NE + CVCC230 group compared to the 23,017 + CVCC230 group. In colon tissues, the 23,017 + CVCC230 (5.38 ± 0.15) group and the 23,017 + NE + CVCC230 (5.95 ± 0.17) group also reduced the number of *E. coli* in comparison to the CVCC230 (6.98 ± 0.16) group, while the level of duodenum tissues in the number of *E. coli* was increased in comparison to the colon tissues, proving that the duodenum may be the main colonization site. We deduced that the addition of NE gives *E. coli* an advantage in the competition with *Levilactobacillus* for colonization sites.

### 3.6 NE attenuated the protective effect of *Levilactobacillus brevis* 23,017 on intestinal tissue integrity

**Figure 5:**
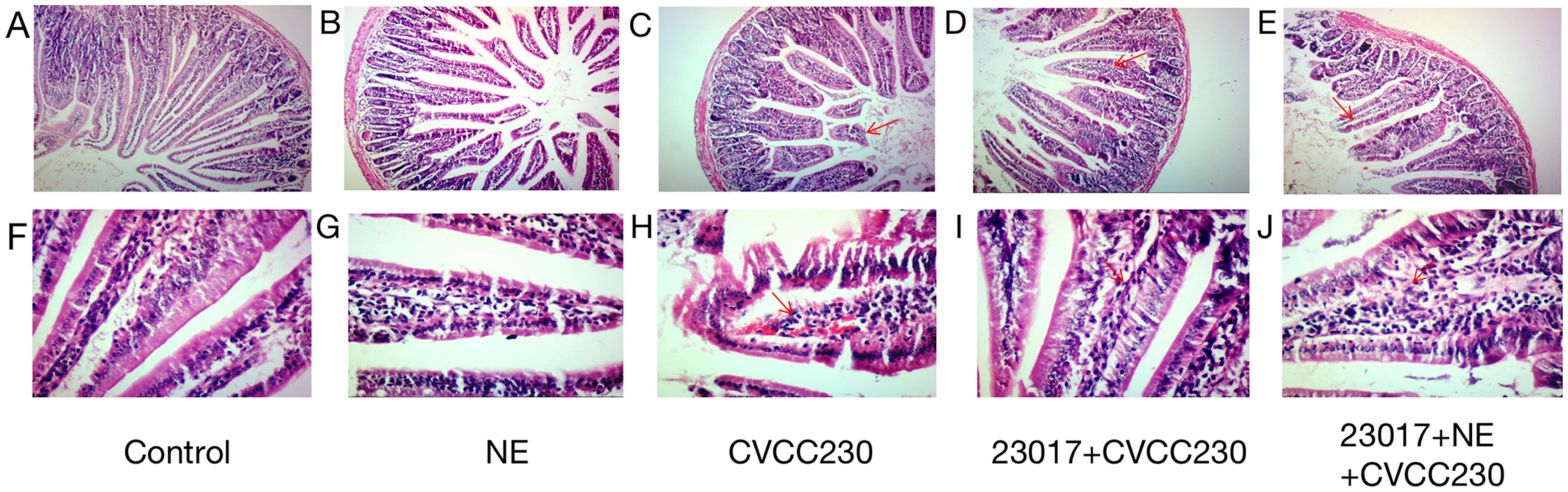
Histological image of duodenum. **(A,F)** Group control; **(B,G)** Group NE; **(C,H)** Group CVCC230; **(D,I)**: Group 23,017 + CVCC230; **(E,J)**: Group 23,017 + NE + CVCC230. **(A–E)** Histological image of duodenum (100×); **(F–J)** Histological image of duodenum (400×). The red arrows in the figure mark the areas where the damage mainly occurs.

To investigate the effect of NE on the capability of *L. brevis* 23,017 to protect the integrity of intestinal tissue, we performed a histological evaluation. As shown in Figure 5, microscopic duodenum damage observed in the CVCC230 group was significantly higher than that in the untreated control and NE groups (Figures 5C,H). There is a large area of tissue shedding, interstitial congestion, and other pathological changes. However, the histopathological signatures were ameliorated by *L. brevis* 23,017 monotherapies (Figures 5D,I), which obviously ameliorated the deterioration of mucosal tissue caused by *E. coli* CVCC230. Moreover, the degree of mucosal inflammation deteriorated in mice that were given the probiotic and NE acting together, with observations of increased mucosal lesions, vasodilation, and increased inflammatory infiltration (Figures 5E,J). That is, the addition of NE reduced the level of cellular integrity protection effect of *L. brevis* 23,017 on intestinal tissues.

### 3.7 NE reduces the antioxidant capacity of *Levilactobacillus brevis* 23,017

To determine whether NE acts on *Levilactobacillus* strains to influence their ability in reducing oxidative stress, we measured some indicators of the antioxidant enzyme system. As shown in Table 1, the CVCC230 group in the mice exhibited significantly elevated SOD (*p* < 0.05), GSH-Px (*p* < 0.05), and T-AOC activity in the duodenum in comparison to the control group. The 23,017 + CVCC230 group resulted in significantly elevated SOD, GSH-Px (*p* < 0.05), and T-AOC activity compared to the CVCC230 group, while the level of MDA is the opposite. At the same time, the levels of SOD (*p* < 0.05), GSH-Px (*p* < 0.05), and T-AOC are significantly reduced, while the level of MDA is elevated in comparison to the 23,017 + CVCC230 group. Taken together, these data show that the addition of NE increases oxidative damage induced by *E. coli* in the organism, indicating that NE decreased the antioxidant capacity of *L. brevis* 23,017.

**Table 1:** Antioxidant indices in duodenum results.

Duodenum	MDA (nmol/mgprot)	SOD (U/mgprot)	GSH-Px (U/mg prot)	T-AOC (U/mg prot)
Control	2.24 ± 1.26	33.9 ± 2.62	484.92 ± 1.56	0.76 ± 0.56
NE	3.43 ± 0.35	19.13 ± 2.4	523.2 ± 2.95	0.49 ± 0.67
CVCC230	5.03 ± 1.16	27.12 ± 0.67^ab**^	452.63 ± 1.83^ab**^	0.48 ± 0.11
23,017 + CVCC230	3. 9 ± 0.95	30.87 ± 3.01^b**^	642.77 ± 1.52^ab**c^	0.57 ± 0.11
23,017 + NE + CVCC230	5.1 ± 2.86	18.92 ± 1.4^acd^	571.37 ± 0.97^ab**cd^	0.35 ± 0.02^c^

^a, b, c^ Mean significant differences between different groups under the same indicator (p < 0.05); “a” means a significant difference between the control and treatment, “b” means a significant difference between the NE and treatment, “c” means a significant difference between the 23,017 + CVCC230,23017 + NE + CVCC230 with CVCC230, “d” means a significant difference between the 23,017 + CVCC230 and 23,017 + NE + CVCC230. **respectively represent significant differences (*p* < 0.01).

### 3.8 NE modulates the ability of *Levilactobacillus brevis* 23,017 in regulating inflammatory factors of serological levels

**Figure 6:**
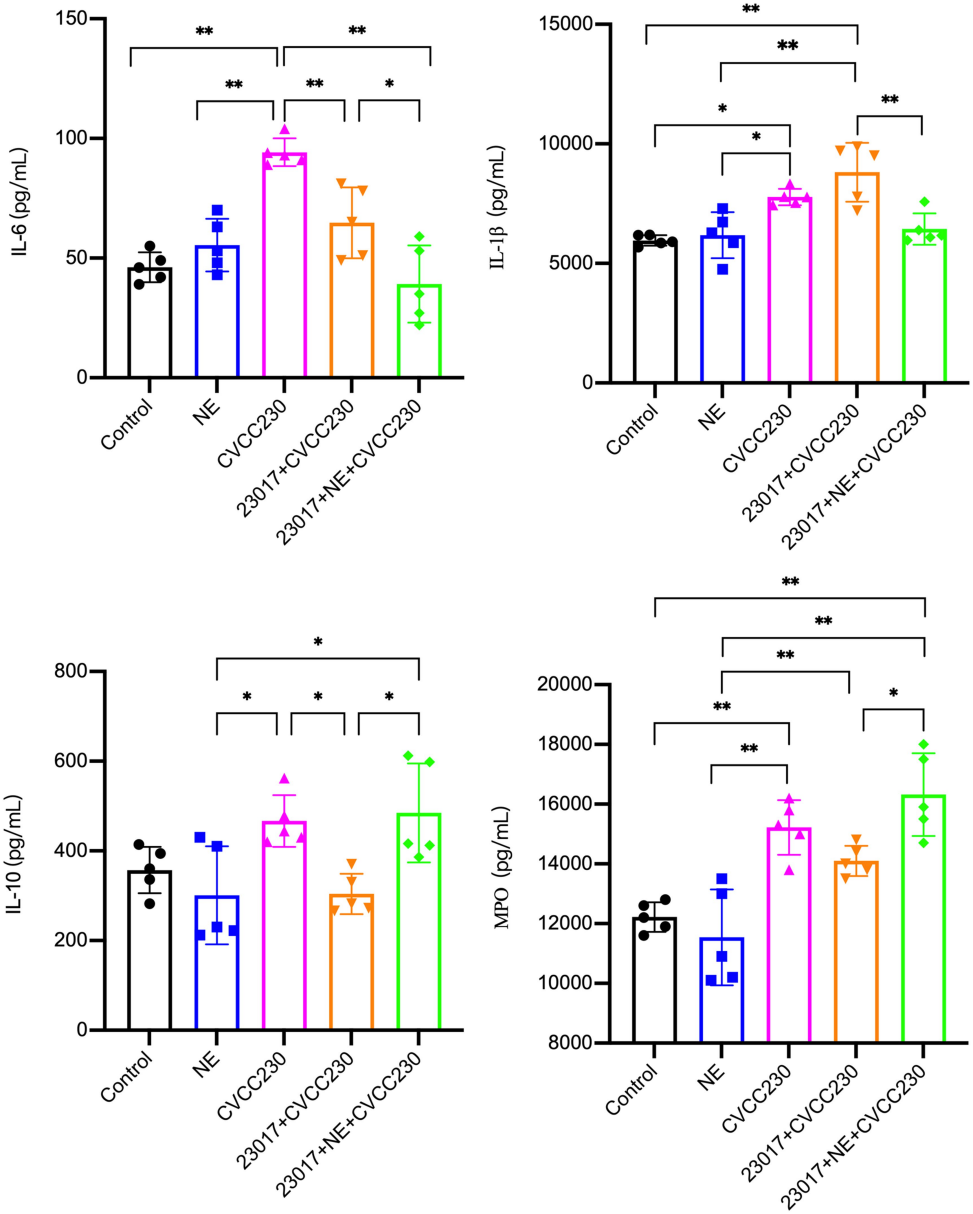
Detection of inflammatory response at serological levels by Elisa method, abscissas represent groups and ordinates represent the concentration of IL-6, IL- IL-1*β*, IL-10 and MPO. Experiments were repeated three times, *, **, respectively, represent significant differences (*p* < 0.05), significant differences (*p* < 0.01).

To reflect the effect of NE on the ability of *Levilactobacillus* in modulating the inflammatory response at serological levels, serum IL-6, IL-1β, IL-10, and MPO were tested (Figure 6). In the CVCC230 group, the expressions of IL-6 (*p* < 0.01), IL-1β (*p* < 0.05), IL-10, and MPO (*p* < 0.01) were upregulated compared to the control group. The mice in the 23,017 + CVCC230 group were found to have downregulated levels of IL-6 (*p* < 0.01), IL-10 (*p* < 0.05), and MPO (*p* < 0.01) compared to the CVCC230 group, with increased expression of IL-1β. The expressions of IL-10 (*p* < 0.05) and MPO (*p* < 0.05) were upregulated, while those of IL-6 (*p* < 0.05) and IL-1β (*p* < 0.01) were downregulated in the 23,017 + NE + CVCC230 group compared to the 23,017 + CVCC230 group. Overall, the addition of NE affects the ability of *L. brevis* 23,017 in modulating the inflammatory response at serological levels.

### 3.9 NE modulates the ability of *Levilactobacillus brevis* 23,017 in regulating inflammatory factors of mRNA levels

**Figure 7:**
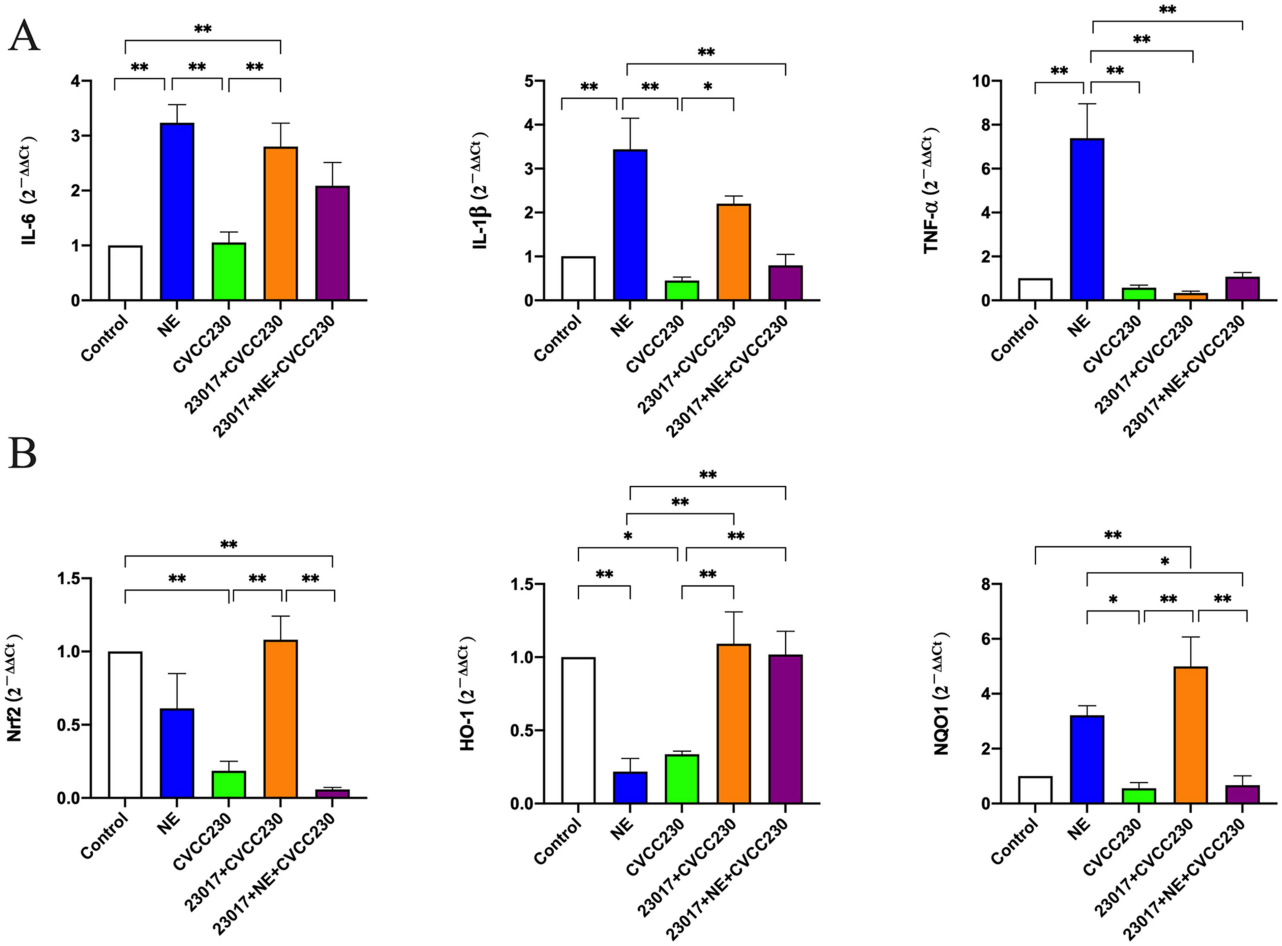
**(A)** Detection of inflammatory response at mRNA levels. **(B)** Detection of mRNA levels of Nrf2 and its downstream genes. Real-time qPCR reactions were used to detect, and β-actin was seen as house-keeping gene. The abscissas represent groups, the ordinates represent 2^−ΔΔCt^ value. Experiments were repeated three times, *, **, respectively, represent significant differences (*p* < 0.05), significant differences (*p* < 0.01).

To reflect the effect of NE on the ability of *Levilactobacillus* in modulating the inflammatory response at mRNA levels, IL-6, IL-1β, and TNF-*α* were tested (Figure 7A). IL-6 (*p <* 0.01), IL-1β (*p* < 0.01), and TNF-α (*p* < 0.01) were all upregulated in the NE-treated group compared to the control group. Compared to the control group, the expressions of IL-1β and TNF-α were downregulated, and the level of IL-6 was unchanged in the CVCC230 group. IL-6 (*p* < 0.01) and IL-1β (*p* < 0.05) expressions were upregulated and TNF-α expression was downregulated in the 23,017 + CVCC230 group compared to the CVCC230 group. Compared to the 23,017 + CVCC230 group, the expressions of IL-6 and IL-1β were downregulated and the expression of TNF-α was upregulated in the 23,017 + NE + CVCC230 group. Overall, the addition of NE affects the ability of *L. brevis* 23,017 in modulating the inflammatory response at mRNA levels.

### 3.10 NE modulates *Levilactobacillus brevis* 23,017 in downregulation of Nrf2 expression

To determine the potential role of NE in the antioxidant effect of *Levilactobacillus*, we detected the mRNA levels of Nrf2 and its downstream genes, an important transcription factor of the oxidative stress response (Figure 7B). As expected, treatment with CVCC230 significantly downregulated the Nrf2 (*p* < 0.01), HO-1 (*p* < 0.05), and NQO1 expressions compared to the control group, while after preventive *Levilactobacillus* treatment, the levels were significantly upregulated (*p* < 0.01). In contrast, preventive NE and *Levilactobacillus* treatments induced downregulation of the Nrf2 (*p* < 0.01), HO-1, and NQO1 (*p* < 0.01) expressions compared to the 23,017 + CVCC230 group. These results show that NE inhibits the expression of Nrf2 and its downstream genes in mRNA levels.

### 3.11 Action of NE on *Levilactobacillus brevis* 23,017 affects mRNA expression levels of functional genes related to intestinal mucosal barrier

**Figure 8:**
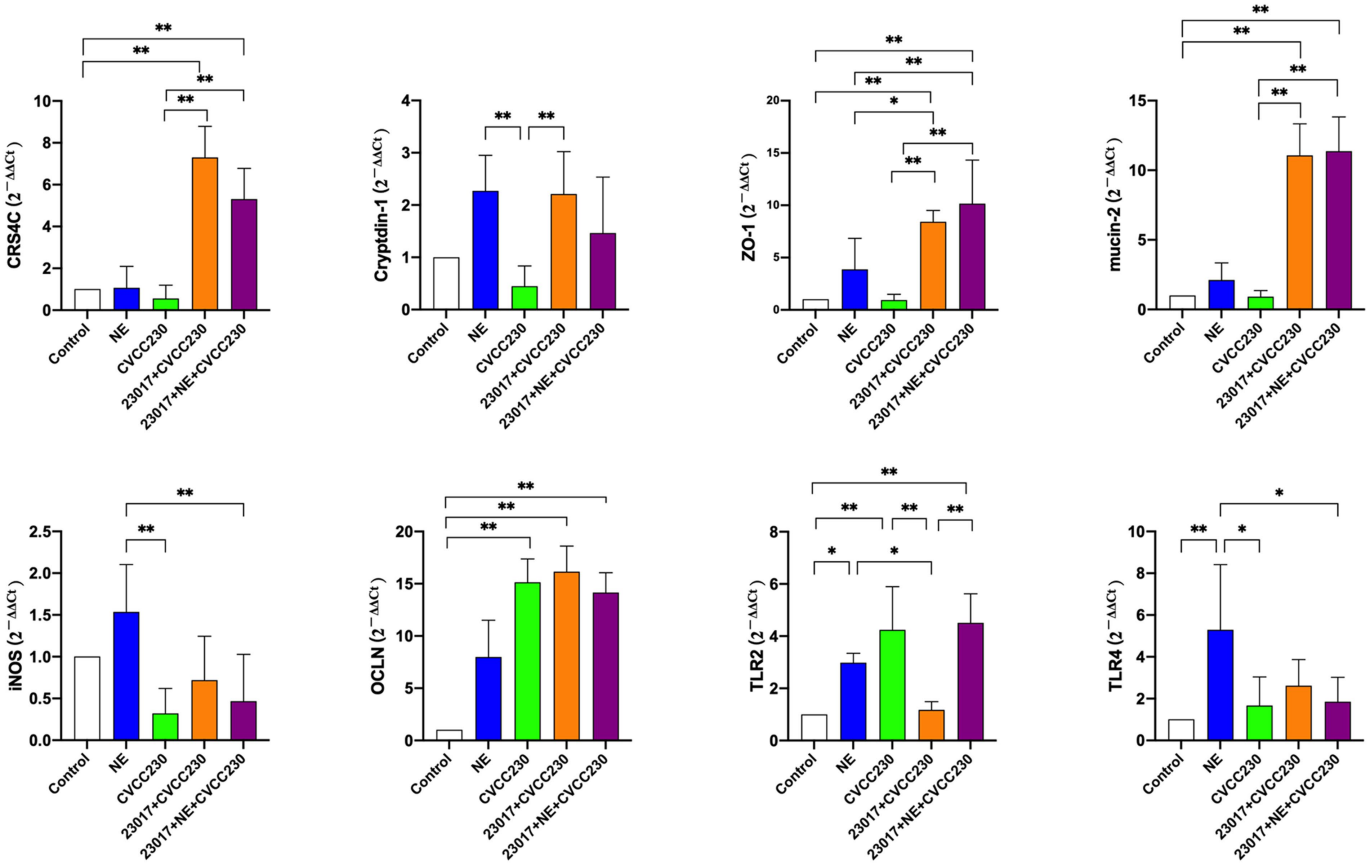
Detection of genes related to the intestinal mucosal barrier at mRNA levels. Real-time qPCR reactions were used to detect, and β-actin was seen as house-keeping gene. The abscissas represent groups, the ordinates represent 2^−ΔΔCt^ value. Experiments were repeated three times, *, **, respectively, represent significant differences (*p* < 0.05), significant differences (*p* < 0.01).

To investigate whether the action of NE on *Levilactobacillus* strains affects their ability to protect the integrity of the intestinal mucosa, we examined the mRNA levels of intestinal mucosal barrier-related genes. As shown in Figure 8, the mRNA expression profiles of CRS4C, Cryptdin-1, ZO-1, mucin-2, and iNOS in the CVCC230 group were decreased when compared to those of control mice, whereas OCLN (*p* < 0.01), TLR2 (*p* < 0.01), and TLR4 were increased. The mRNA expression profiles of CRS4C (*p* < 0.01), Cryptdin-1 (*p* < 0.01), ZO-1 (*p* < 0.01), mucin-2 (*p* < 0.01), OCLN, TLR4, and iNOS in the 23,017 + CVCC230 group were significantly elevated when compared to the CVCC230 group, while TNF-*α* and TLR2 (*p* < 0.01) expressions were downregulated. Compared to the 23,017 + CVCC230 group, the expressions of ZO-1, mucin-2, and TLR2 (*p* < 0.01) in the 23,017 + NE + CVCC230 group were increased, while the expressions of CRS4C, Cryptdin-1, OCLN, TLR4, and iNOS were decreased. In conclusion, NE affects the ability of *L. brevis* 23,017 in enhancing the expression of intestinal mucosal barrier-related genes.

### 3.12 NE affects the ability of *Levilactobacillus* in maintaining the balance of the intestine bacterial microbiota composition

**Figure 9:**
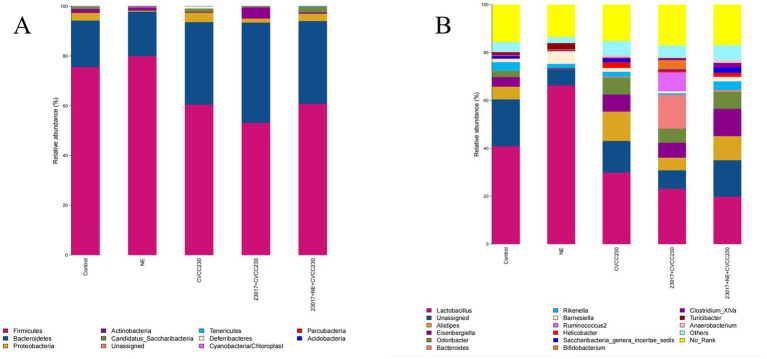
**(A)** The intestinal microbiota at the phylum level were analyzed using 16S rRNA sequencing results. The horizontal axis represents the sample, and the vertical axis represents the relative richness value. **(B)** The intestinal microbiota at the genus level were analyzed using 16S rRNA sequencing results. The horizontal axis represents the sample, and the vertical axis represents the relative richness value.

To investigate whether NE affects the function of *Levilactobacillus* in regulating intestinal health, we performed an analysis of the microbial composition of the intestinal flora (Figure 9). The 11 dominant bacterial phyla (with mean relative abundance >1%) are shown in Figure 9A, and at the genus level (Figure 9B), 16 dominant bacterial genera (average relative abundance >1%) could be identified. In comparison of the relative abundance of *Levilactobacillus* in each group, the relative abundance of *Levilactobacillus* was the highest in the NE group, followed by the normal control group. The members of the *Levilactobacillus* family were in low abundance in the 23,017 + NE + CVCC230 group as compared to the 23,017 + CVCC230 group. From the above results, we can see that NE induces an increase in the relative abundance of probiotics in the intestinal flora, and oral administration of NE affects the balance of intestinal flora.

### 3.13 NE modulates the ability of *Levilactobacillus* in maintaining the balance of species richness and diversity in intestinal flora

To investigate whether NE affects the function of *Levilactobacillus* in regulating intestinal flora species diversity, we measured the richness and diversity of the intestine’s bacterial microbiota. The α diversity indices of the samples are shown in Table 2. The values of Chao1, ACE, and Simpson were increased in the CVCC230 and 23,017 + CVCC230 groups compared to the control group, while the value of Shannon was decreased. The values of Chao1, ACE, and Shannon in the 23,017 + NE + CVCC230 group were increased when compared to the 23,017 + CVCC230 group, while Simpson was decreased. There is no significant difference between each group. Overall, this result indicates that the 23,017 + NE + CVCC230 group had the highest species richness and the highest community diversity.

**Table 2:** Results of intestinal microflora abundance and diversity in mice.

Group	Chao1	ACE	Shannon	Simpson
Control	346.991 ± 38.28	347.005 ± 65.43	3.375 ± 2.43	0.074 ± 0.012
NE	224.072 ± 22.34	238.287 ± 64.11	2.566 ± 0.94	0.153 ± 0.10
CVCC230	371.317 ± 83.2	371.066 ± 83.62	3.375 ± 1.43	0.102 ± 0.05
23,017 + CVCC230	397.665 ± 43.43	382.862 ± 32.98	3.286 ± 1.11	0.082 ± 0.02
23,017 + NE + CVCC230	412.712 ± 21.11	404.980 ± 73.02	3.950 ± 0.83	0.048 ± 0.01

## 4 Discussion

Microbial endocrinology is a relatively young scientific field, and for decades, scientists have studied the activity of catecholamines only in terms of host immune response, vasoconstriction, and so on. The direct effects on bacteria have been explored mostly in pathogenic bacteria and very few in probiotics (Boukerb et al., 2021). Many clinical diseases are induced by enterotoxigenic *E. coli* (ETEC), and several stress hormones, especially catecholamines, can aggravate the condition and cause anorexia, intestinal inflammation, and intestinal flora imbalance (McCracken et al., 1999; Bomba et al., 2014). Currently, treatment for *E. coli* infection relies primarily on antibiotics, but they can cause many side effects. *As an antibiotic alternative*, *Levilactobacillus* is extremely widespread in nature and plays a vital role in resistance to pathogen infection and keeping the gastrointestinal tract healthy (Round and Mazmanian, 2009; Liu et al., 2014; Ritze et al., 2014; Dowarah et al., 2017; Vinasco et al., 2019). However, it has different effects in different individuals, or in laboratory and clinical applications. Therefore, in this study, we tried for the first time to analyze the effects of NE on the probiotic properties of *Levilactobacillus* strains *in vivo* to resolve the inconsistent effect of the application of *Levilactobacillus*. This study fills some of the gaps in microbial endocrinology with respect to probiotics and is important for both the theoretical basis and clinical application of the study of stress hormone interactions with probiotics.

When the *Levilactobacillus* strains were tested *in vitro* in the presence of NE, 80% of the strains showed an increase in growth rate and the number of viable bacteria. This is in general agreement with majority of the previous studies of pathogenic Gram-positive bacteria (Belay and Sonnenfeld, 2002; Lyte et al., 2003; Sandrini et al., 2014; Boyanova, 2017). We speculate that NE-induced growth of *Levilactobacillus* strains may be related to iron homeostasis and gene transcription. The study by Bearson et al. indicated that *Salmonella Typhimurium* changes the biosynthesis of numerous cellular pathways to increase its growth rate in serum-SAPI minimal media to utilize the increased availability of iron provided by NE (Bearson et al., 2008; Boukerb et al., 2021). Iron sequestration is mainly due to the mammalian ferric-iron-binding proteins transferrin in serum and lactoferrin in mucosal secretions (Mietzner and Morse, 1994). Many studies demonstrating the influence of catecholamines on bacterial growth used a serum-like medium containing transferrin (Tf) and lactoferrin (Lf). Serum-SAPI minimal media more accurately simulate a stressful and bacteriostatic environment for the bacteria, and so more precisely they resemble the conditions that the bacteria may experience within the host (Mietzner and Morse, 1994; Freestone et al., 2003; Lyte, 2004). In several other studies, NE inhibits the growth of *Prevotella intermedius* (Jentsch et al., 2013) and *Porphyromonas pulposus* (Calil et al., 2014). The difference between promotion and inhibition may be related to the concentration of hormones (Roberts et al., 2002; Hegde et al., 2009; Jentsch et al., 2013; Calil et al., 2014), but we only selected the optimal concentration for investigation based on the clues provided by previous experiments. In the next step, we selected representative numbers of concentrations to investigate. Overall, our study demonstrates that NE has a beneficial effect on the growth of *Levilactobacillus* strains, and this study fills the gaps in microbial endocrinology in the study of probiotic *Levilactobacillus* strains.

The antimicrobial activity of more than 80% of the *Levilactobacillus* strains against three indicator bacteria at 22 h was decreased due to the presence of NE. This study investigates the effect of catecholamine hormones on the antimicrobial activity of probiotic *Levilactobacillus* strains. According to the results of the preliminary pre-experiment, we found that the effect of *Levilactobacillus* strains on *E. coli*, *S. aureus*, and *P. aeruginosa* was stronger, so we explored the effect of NE on the antimicrobial activity of *Levilactobacillus* strains using three indicator bacteria in this experiment. Metabolites of *Levilactobacillus* strains such as organic acids, hydrogen peroxide, antimicrobial peptides, and bactericidal proteins are the main substances that exert antibacterial effects (Awaisheh and Ibrahim, 2009; Gupta and Srivastava, 2014). Some studies have shown that catecholamines can play a role in free radical generation (Borisenko et al., 2000), and bacteria may promote adrenaline oxidation to adrenochrome and produce superoxide (Toulouse et al., 2019; Reiske et al., 2020). We hypothesized that if NE also reacted in this way, it would affect the probiotic viability of *Levilactobacillus.* We imply that NE may reduce the antibacterial effect of *Levilactobacillus* strains by affecting their metabolites. The results provide a theoretical basis for the next application of *Levilactobacillus* in practice.

By simulating the coexistence of three factors in the intestine *in vivo*, the interaction increases the number of viable bacteria of *E. coli*. Our previous study has shown that NE promotes the growth of *E. coli* CVCC230. Although we found that NE inhibited the anti-*E. coli* ability of *Levilactobacillus* strains and significantly increased the viable *E. coli* count in the three-factor mixed environment, it was impossible to conclude that NE reduced the antimicrobial activity of *Levilactobacillus* against *E. coli* CVCC230 in this environment as the competition for nutrients in the medium could also contribute to the higher counts. The role of NE in the organism is very complex and needs to be analyzed from multiple perspectives. Our discussion of the coexistence of the three factors in the intestine is not comprehensive enough, and we wish to provide clues for the next studies in the microbial endocrinology field, *in vitro* or *in vivo*.

In the presence of NE, the acid production capacity of 60% *Levilactobacillus* strains was enhanced without any effect on the hydrogen peroxide production capacity. Past studies have shown that the primary antimicrobial activity of certain strains depends on the production of acid or hydrogen peroxide (Blajman et al., 2015). However, we speculate that the diminished antimicrobial activity of NE on *Levilactobacillus* strains is not acting through these two characteristics in this study. NE probably reduces the antimicrobial capacity of *Levilactobacillus* strains by affecting the secretion of antimicrobial peptides or antimicrobial compounds (Awaisheh and Ibrahim, 2009; Ren et al., 2018). We attempted to elucidate the mechanism of NE action on *Levilactobacillus* strains through *in vitro* experiments, and our study provides a basis for the exploration of microbial endocrinology in *Levilactobacillus*.

The results of intestinal colonization and observation of pathological sections demonstrated that giving NE and *L. brevis* 23,017 together attenuated the inhibitory effect of *E. coli* CVCC230 colonization and increased mucosal lesions, vasodilatation, and inflammatory infiltration relative to the group given *L. brevis* 23,017 alone. The study of Cambronel et al. showed that catecholamine hormones influenced the adhesion of both pathogenic and probiotic *Enterococcus faecalis* (Cambronel et al., 2020). It has also been suggested that catecholamine hormones may have opposite effects on the ability of different pathogenic bacteria to adhere (Gonzales et al., 2013). We speculate that NE may have affected the adhesion and competition for attachment sites of *L. brevis* 23,017 and weakened its inhibitory effect on *E. coli* colonization. Previously, it was investigated that NE at the cellular level enhanced the probiotic properties of *Enterococcus faecalis*, such as resistance to bile salts, autoaggregation, and biofilm formation (Scardaci et al., 2021). However, our results demonstrate that NE negatively regulates the probiotic effect of *L. brevis* 23,017. In the context of the microbiota–gut axis communication, the activities of the organism at all levels are complex (Lyte, 2016), and our study is important to elucidate the interactions between symbiotic bacteria and their hosts.

The detection of oxidative stress-related markers concluded that NE decreased the antioxidant capacity of *L. brevis* 23,017. Many past studies have indicated that treatment with *Levilactobacillus* showed higher antioxidant capacity than that of other groups; the same was true for us (Ge et al., 2021). Previous studies have shown that the autotrophic antioxidant enzyme systems, chelated metal ions, and regulation of the intestinal bacteria group are the primary ways that the antioxidant mechanism of *Levilactobacillus* is exhibited (Li et al., 2012). We speculate that NE may also affect the antioxidant capacity of *L. brevis* 23,017 in these ways, and we intend to continue investigating the mechanisms at the gene level or signaling pathway in the next step. Overall, this again confirms that NE can reduce the probiotic effects of *Levilactobacillus* strains.

The assay of inflammatory factors at serological levels showed that NE acting on *L. brevis* 23,017 may promote an anti-inflammatory environment by downregulating the IL-6 and IL-1β levels and upregulating the levels of IL-10 and MPO transcripts. The NE group had significantly higher levels of inflammatory factor mRNA expression, and the relative tendency in the 23,017 group was completely opposite to that in the 23,017 + NE group, demonstrating that the addition of NE inhibited the regulation of intestinal inflammatory factors by *L. brevis* 23,017. The results obtained from our assay of serum and intestinal levels of inflammatory factors did not show a significant trend toward consistency. As reported in a previous study, Mu et al. indicated that the transcriptional level was not consistent in the intestine, spleen, and serum with *Levilactobacillus* treatment, suggesting gut-specific immunosuppression (Mu et al., 2017). Even though in some circumstances IL-6 signaling has been characterized as anti-inflammatory, it also plays a crucial role in increasing inflammation and immunity (Hirano et al., 2000; Vardam et al., 2007; Silver and Hunter, 2010; Rose-John, 2012). Moreover, many pro-tumor actions can be supported by stimulation of IL-6 trans signaling, which also has the ability to boost adaptive immunity against tumors (Fisher et al., 2014). Together, these findings suggest that NE has a role in the ability of *L. brevis* 23,017 to modulate the inflammatory response, but the exact pattern of action still needs to be explored.

In the mouse duodenum, NE on *L. brevis* 23,017 decreased Nrf2 and its downstream gene expression. Nrf2 is a transcription factor that helps regulate the cellular oxidative stress (Ma, 2013). It is an important regulator of cellular defense mechanisms against xenobiotics and oxidative stress (Tonelli et al., 2018). As a part of the gut microbiota, *Levilactobacillus* benefits in regulating oxidative stress in tissues and cells, and the results of Long et al. (2021) are similar to ours and show that *L. fermentum* can also promote high-level expression of Nrf2 and other downstream antioxidant factors such as HO-1 and NQO1. Previous research has shown that Nrf2 signaling pathways mediate the defense mechanisms against oxidative stress and inflammation (Fuentes et al., 2015; Li et al., 2018). Therefore, we speculate that NE acting on *L. brevis* 23,017 may reduce the antioxidant effect by regulating the expression of Nrf2 and its downstream genes then affect their protein expression. The addition of NE reduced the *L. brevis* 23,017 enhancement of intestinal mucosal barrier-related gene expression *in vivo* in the mouse. Catecholamines, particularly NE, are abundantly present in the intestinal mucosa during stressful conditions (Eisenhofer et al., 2004). Previous research has demonstrated that *Levilactobacillus* can improve intestinal mucosal status and increase the expression of intestinal mucosal barrier-associated proteins (Chaudière and Ferrari-Iliou, 1999; Mao et al., 2016), which is consistent with our study. However, NE acting on *L. brevis* 23,017 downregulated the expression of antimicrobial peptides CRS4C and Cryptdin-1 and the tight junction protein OCLN. We speculate that NE affects the probiotic effects of *L. brevis* 23,017 by affecting its ability to regulate intestinal mucosal secretion.

The effect of oral NE modulates the balance of intestinal flora, causing a decrease in the relative quantity of probiotic bacteria and an increase in the relative abundance of other harmful bacteria. NE may act on numerous gut microorganisms. According to the *in vitro* part of this study, NE attenuated the inhibitory effects of *L. brevis* 23,017 on *E. coli*, *S. aureus*, and *P. aeruginosa*. Since previous studies have shown that catecholamine hormones have a promoting effect on *E. coli* growth and virulence, we hypothesize that one of the reasons why the addition of NE causes an imbalance in the intestinal flora may be due to a reduction in the inhibitory effect of *Levilactobacillus* on the harmful microorganisms in the intestinal tract. The development and changes of the intestinal microbiota have a non-negligible impact on the development and treatment of many diseases (Linninge et al., 2019). Previous studies have shown that *Levilactobacillus* strains can regulate the disease process by maintaining the balance of intestinal flora (Mu et al., 2017). We suggest that the imbalance of intestinal flora regulated by NE action on *Levilactobacillus* and the change of intestinal flora will also regulate the immune response of the body, leading to the occurrence of stress, creating a vicious circle until it affects systemic immunity. The highest relative abundance of *Levilactobacillus* was obtained by the assay of intestinal flora in the NE group, followed by the normal control group. This was validated against the promotion of bacterial growth by *in vitro* levels of NE, demonstrating that NE also enhanced the growth of *Levilactobacillus* under complex conditions in the *in vivo* organism. Overall, the addition of NE modulates the balance of intestinal flora.

This study was only a basic one hoping to provide some clues for microbial endocrinology. Owing to the complexity of NE activity in the organism and the fact that the addition of NE may also affect some other gut microorganisms in the organism, we have examined only the changes in the organism that may be induced by the concomitant administration of NE and *L. brevis*. Hopefully, some evidence can be given that in animals, the addition of norepinephrine also reduces the probiotic effect of lactobacilli on the body. Studies have shown that catecholamine receptor antagonists are therapeutically important in the treatment of conditions such as hypertension, and adrenergic and dopaminergic antagonists block NE, epinephrine, and Dopa responses in bacteria (Freestone et al., 2008). Whether we can prevent the disease by blocking the catecholamine hormones in the body is the next major direction of our research. In the next step, we will use this study as a basis to investigate the effects of different types of receptor inhibitors and catecholamine hormones under different environmental conditions and adjust the assay indicators and methods to carry out more in-depth studies.

## 5 Conclusion

In conclusion, this is the first report on how probiotics and catecholamine hormones interact in a mouse model. The results showed that NE promoted growth while suppressing the antimicrobial capacity of *Levilactobacillus* strains, and the viable count of *E. coli* was increased in the presence of *Levilactobacillus* strains and neurohormone coexistence. In addition, the administration of NE in the ETEC-infected mouse model reduced the ability of *L. brevis* 23,017 in inhibiting pathogenic bacterial colonization of the intestine, inhibiting intestinal inflammatory cell infiltration, antioxidant capacity, protection of the intestinal mucosal barrier, and maintaining intestinal flora homeostasis. Our study is the first report to investigate the effect of catecholamine hormones on the properties of probiotics *in vivo* and may provide a new direction to address the unstable clinical application of *Levilactobacillus* strains and improve the application of *Levilactobacillus* as an antibiotic alternative.

## Data Availability

The original contributions presented in the study are publicly available. This data can be found here: www.ncbi.nlm.nih.gov, accession number PRJNA1212890.
