# Supplementary material for: Effects of the stress hormone norepinephrine on the probiotic properties of Levilactobacillus: antibacterial colonization, anti-inflammation, and antioxidation
Source: Front Microbiol. 2025 Feb 10;16:1526362. doi: 10.3389/fmicb.2025.1526362 (PMC11849050; doi:10.3389/fmicb.2025.1526362)
Supplement: Supplementary file 1 [file Table_1.DOCX]

Supplementary Material

**Table S1** PCR primers sequences.

| Gene | Primer | Sequence (5'–3') |
| --- | --- | --- |
| β-actin | β-actinF | CTGGAACGGTGAAGGTGA |
|  | β-actinR | TTTGGAAAGGCAGGGACT |
| IL-6 | IL-6F | AAAGAGTTGTGCAATGGCAATTCT' |
|  | IL-6R | AAGTGCATCATCGTTGTTCATACA |
| IL-1β | IL-1βF | TCTCCAGCCAGTCTTCATTGT |
|  | IL-1βR | GCCATCAGCCTCAAATAACAG |
| TNF-α | TNF-αF | ACCAGCCAGGAGAGAGACAAG |
|  | TNF-αR | AGCGTGTGAGAGGGAGAGAGT |
| TLR2 | TLR2 F | GGGGTGTGTGATGGCCGCTC |
|  | TLR2R | TGGAGGTTCGCACACGCTCG |
| iNOS | iNOS-F | CAGCTGGGCTGTACAAACCTT |
|  | iNOS-R | CATTGGAAGTGAAGCGTTTCG |
| Nrf2 | Nrf2F | GGGGTAAGAATAAAGTGGCTGCTC |
|  | Nrf2R | ACATTGCCATCTCTTGTTTGCTG |
| HO-1 | HO-1F | AGCTGTTTCTGAGCCTCCAA |
|  | HO-1R | CAAGACGGAAACACGAGACA |
| NQO1 | NQO1F | TGCCTTCCTTGACTTGCT |
|  | NQO1R | TCCCGGCTTTACATCCTA |
| CRS4C | Crs4cF | GCATGGAATCTGGGTCAAGATAAC |
|  | Crs4cR | AGAAGGAAGAGCAATCAAGGCTAAG |
| Cryptdin-1 | Cryptdin-1F | CTAGTCCTACTCTTTGCCCT |
|  | Cryptdin-1R | TTGCAGCCTCTTGATCTACA |
| ZO-1 | ZO-1F | AGGACACCAAAGCATGTGAG |
|  | ZO-1R | GGCATTCCTGCTGGTTACA |
| Mucin 2 | Mucin 2F | CCCAGAAGGGACTGTGTATG |
|  | Mucin 2R | TTGTGTTCGCTCTTGGTCAG |
| OCLN | OCLNF | GCTGTGATGTGTGTGAGCTG |
|  | OCLNR | GACGGTCTACCTGGAGGAAC |

**Table S2** Results of relative abundance of phylum level intestinal flora.

| Group | Firmicutes | Bacteroidetes | Proteobacteria | Actinobacteria | Candidatus_Saccharibacteria |
| --- | --- | --- | --- | --- | --- |
| control | 0.7494±0.09 | 0.1821±0.07 | 0.0399±0.01 | 0.0155±0.006 | 0.0093±0.004 |
| NE | 0.8001±0.15 | 0.1782±0.04 | 0.0043±0.002 | 0.0171±0.003 | 0.004±0.001 |
| CVCC230 | 0.5954±0.26 | 0.3305±0.17 | 0.0392±0.009 | 0.0039±0.001^ab^ | 0.0153±0.002 |
| 23017+CVCC230 | 0.5321±0.22 | 0.4027±0.11^a^ | 0.0156±0.005 | 0.0452±0.015 | 0.0031±0.001^c^ |
| 23017+NE+CVCC230 | 0.6084±0.03 | 0.3317±0.18 | 0.0296±0.01^b^ | 0.0061±0.001^d^ | 0.0218±0.007 |

Note：“a, b, c”mean significant differences between different groups under the same indicator (*p* < 0.05);“a” means a significant difference between the control and treatment, “b” means a significant difference between the NE and treatment, “c” means a significant difference between the 23017+CVCC230,23017+NE+CVCC230 with CVCC230, “d” means a significant difference between the 23017+CVCC230 and 23017+NE+CVCC230.

**Table S3** Results of relative abundance of genus level intestinal flora.

| Group | Lactobacillus | Barnesiella | Helicobacter | Bifidobacterium |
| --- | --- | --- | --- | --- |
| control | 0.40862±0.015 | 0.01386±0.009 | 0.00364±0.003 | 0.00257±0.001 |
| NE | 0.66249±0.16 | 0.08010±0.02 | 0 | 0.00615±0.003 |
| CVCC230 | 0.29864±0.19 | 0.01548±0.01 | 0.03621±0.015 | 0 |
| 23017+CVCC230 | 0.23066±0.09 | 0.00892±0.004^ac^ | 0.01218±0.005^a^ | 0.03974±0.014 |
| 23017+NE+CVCC230 | 0.19817±0.07 | 0.01869±0.006 | 0.01718±0.007 | 0.00012±0.0001^d^ |

Note：“a, b, c”mean significant differences between different groups under the same indicator (*p* < 0.05);“a” means a significant difference between the control and treatment, “b” means a significant difference between the NE and treatment, “c” means a significant difference between the 23017+CVCC230,23017+NE+CVCC230 with CVCC230, “d” means a significant difference between the 23017+CVCC230 and 23017+NE+CVCC230.


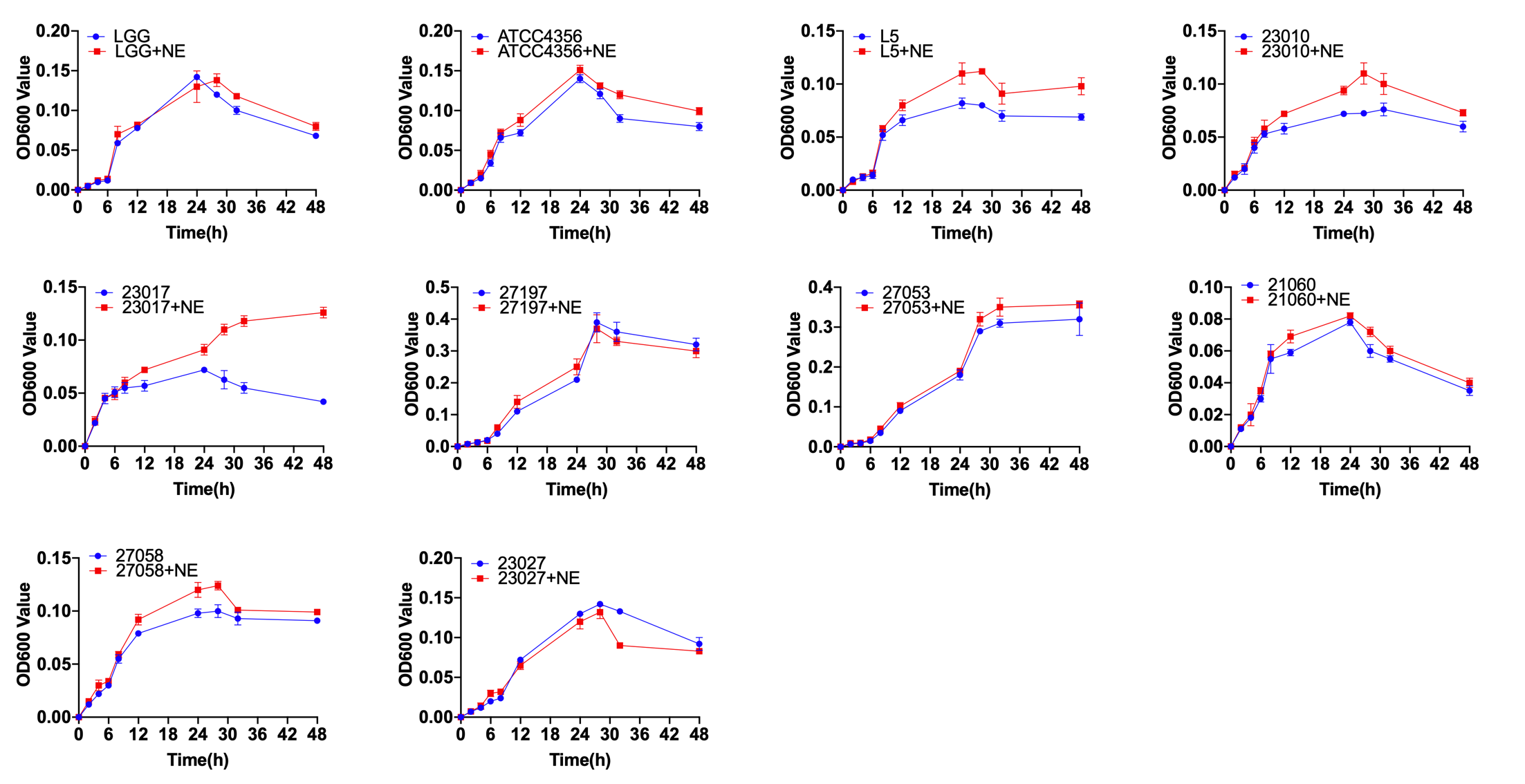


Fig. S1. The effect of norepinephrine (NE) on the growth of *Levilactobacillus*, vertical coordinates are expressed as OD600 values.


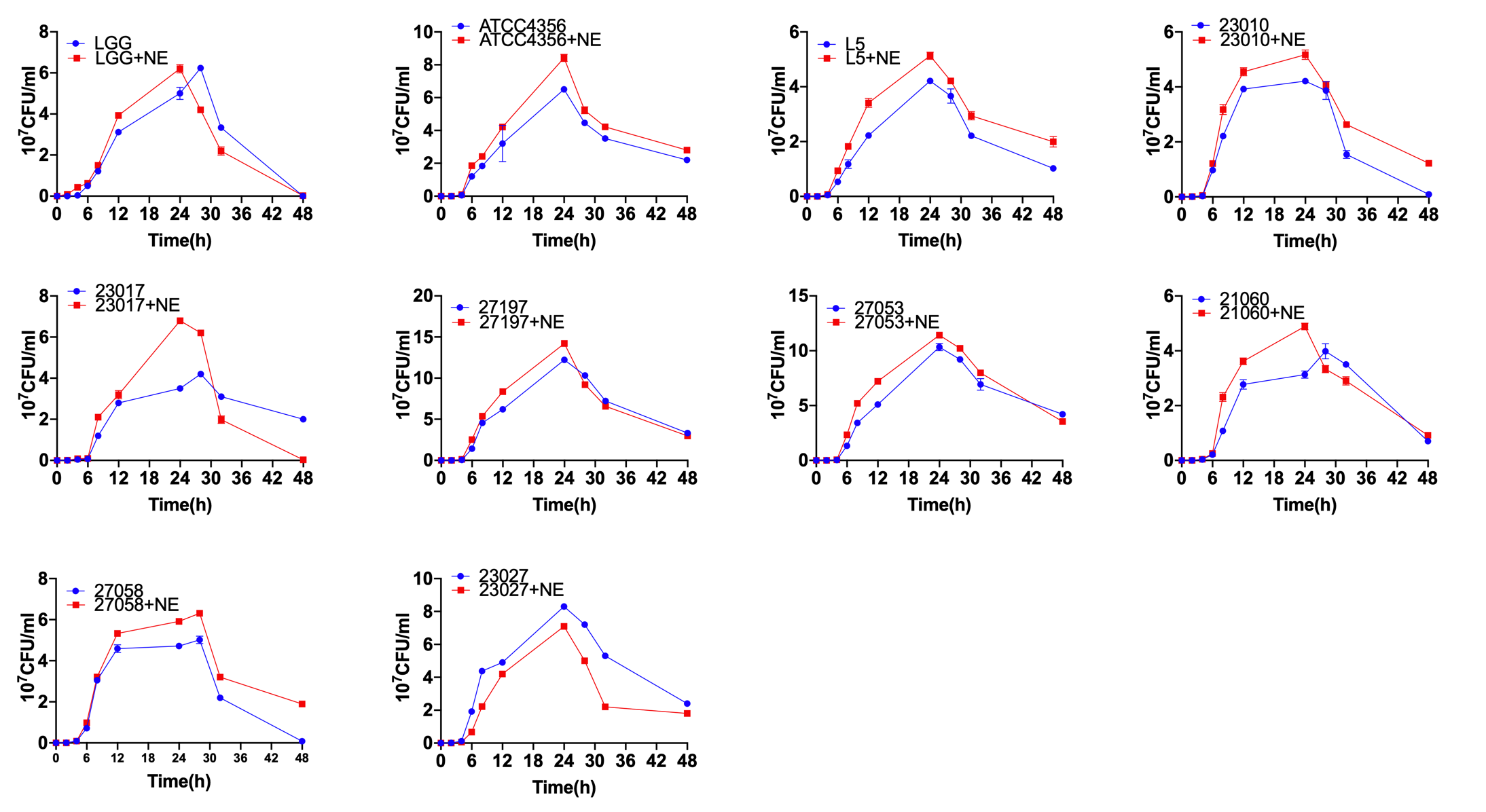


Fig. S2. The effect of norepinephrine (NE) on the growth of *Levilactobacillus*, vertical coordinates are expressed as 10^7^CFU/mL.


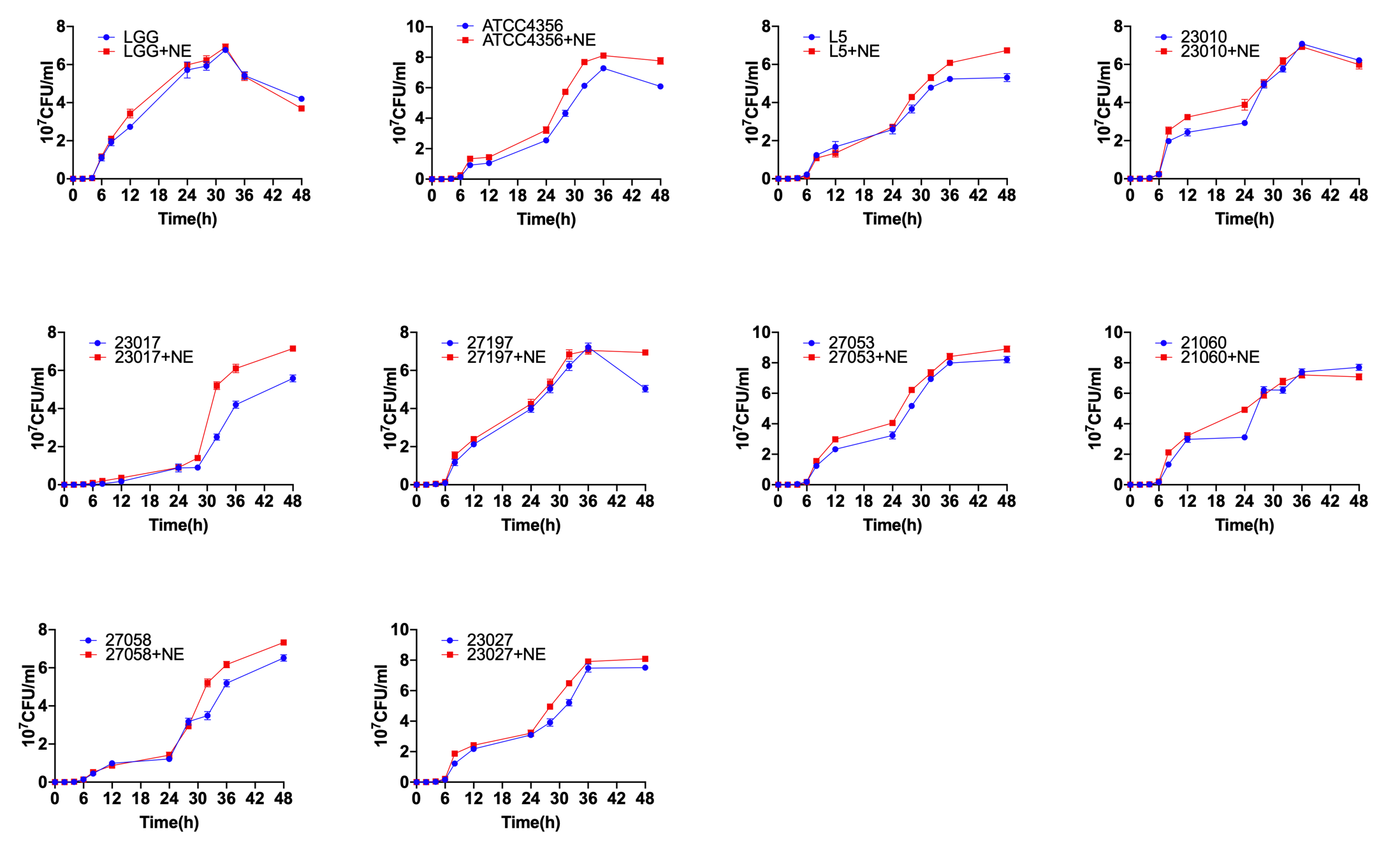


Fig. S3. Effect of NE on the number of viable *Escherichia coli* bacteria under co-culture conditions, vertical coordinates indicate the number of viable bacteria of *E. coli* (10^7^ CFU/mL).


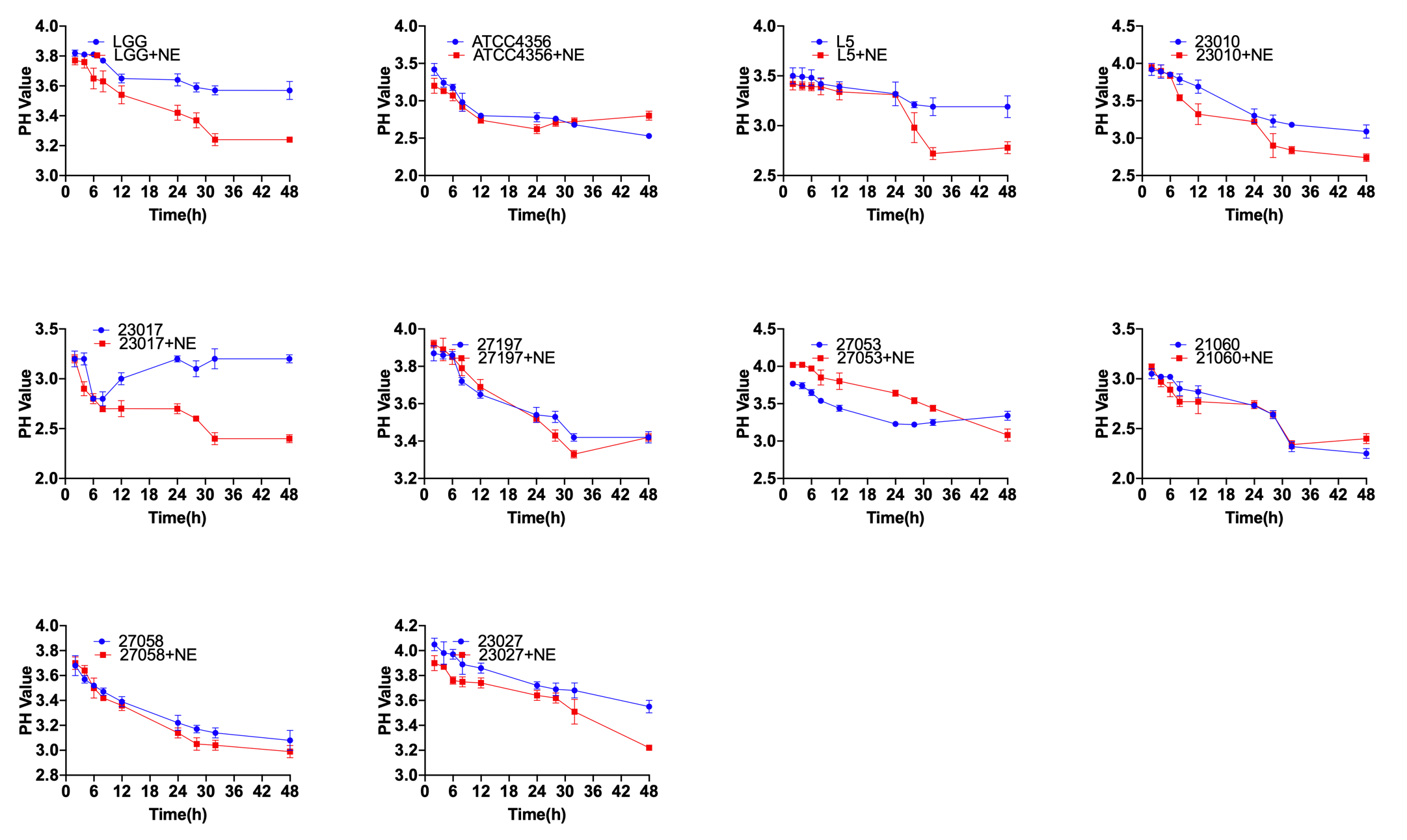


Fig. S4. Effect of NE on acid production of *Levilactobacillus.*
